# Spontaneous Heparin-Induced Thrombocytopenia Following Orthopedic Surgery

**DOI:** 10.7759/cureus.99312

**Published:** 2025-12-15

**Authors:** Aleksey Korolyov, Rong Hu

**Affiliations:** 1 Department of Medicine, University of California Los Angeles, Los Angeles, USA

**Keywords:** deep vein thrombosis (dvt), heparin-induced thrombocytopenia (hit), thrombo embolic disease, thrombosis, total joint arthroplasties

## Abstract

Heparin-induced thrombocytopenia (HIT) is a life-threatening complication most commonly associated with exposure to heparin. Timely recognition of suspected HIT is vital, as early intervention is associated with improved outcomes and a decreased mortality rate. Spontaneous HIT is a rare form that occurs in the absence of heparin exposure. Here, we report the case of an 83-year-old patient diagnosed with spontaneous HIT after undergoing knee replacement surgery.

## Introduction

Heparin-induced thrombocytopenia (HIT) is a complication most commonly associated with exposure to heparin. It results from an autoantibody against endogenous platelet factor 4 (PF4) in complex with heparin, which then activates platelets and can cause arterial and venous thrombosis as well as thrombocytopenia. HIT has been reported in up to 5% of patients exposed to heparin for more than four days. Factors that increase the frequency of HIT include surgery [1], exposure to unfractionated heparin [2], heparin dose (a higher incidence in patients receiving a therapeutic dose than in those receiving a prophylactic dose) [3], female sex [1], and older age [4].

Clinical manifestations of HIT include thrombocytopenia (platelet count < 150,000, mean nadir of 60,000), bleeding, and thrombosis (venous more common than arterial). Given that the results of the immunoassay and functional assay for HIT Ab may not be available for days, the pretest probability of HIT may be established based on clinical findings and laboratory data utilizing the 4Ts score. Parameters of the 4Ts score include thrombocytopenia (% platelet decrease, nadir), timing of onset after heparin exposure, thrombosis or other clinical sequelae, and other causes of thrombocytopenia. Patients with a 4Ts score of intermediate or high probability should have a presumptive diagnosis of HIT. In these patients, HIT Ab testing (immunoassay or functional assay) should be performed to confirm or exclude the diagnosis; heparin should be immediately discontinued, and a non-heparin anticoagulant should be initiated unless there is a contraindication, such as bleeding or a high risk of bleeding. In rare cases, HIT has been diagnosed in patients without recent heparin exposure. Most commonly, these patients have had a preceding infection or have undergone a surgical procedure (particularly orthopedic surgery). This condition has been called "spontaneous HIT" or "autoimmune HIT."

## Case presentation

An 83-year-old female patient with a past medical history of hypertension, hyperlipidemia, type 2 diabetes mellitus, and cognitive impairment presented with unilateral right lower extremity swelling and ecchymoses. Twelve days prior to the presentation, she had undergone right total knee arthroplasty without complication. She was started on aspirin (81 mg po) twice daily for DVT prophylaxis and was compliant with the medication. She worked with physical therapy after surgery and was discharged to a skilled nursing facility for further rehabilitation. She presented to the orthopedic surgery clinic for routine postoperative evaluation, where she was noted to have right leg swelling and surrounding ecchymoses. Her vital signs were normal: she was afebrile, with a normal heart rate and blood pressure, and oxygen saturation was normal on room air. A physical exam revealed the presence of a Prevena wound vac over the right knee, ecchymoses over the lateral knee and leg, and swelling affecting the right leg. Table 1 presents the laboratory values.

**Table 1:** Laboratory values WBC: white blood cell; MCV: mean corpuscular volume

Hematology	7 Days Prior	Admission (Day 0)	Day 15	Day 52	Reference Range
WBC	4.15	6.53	5.25	7.57	4.16–9.95 x 10^3^/µL
Hemoglobin	9	9.7	9.8	12.6	11.6–15.2 g/dL
MCV	94.3	94.5	96.7	93.9	79.3–98.6 fL
Platelet Count	116	22	149	241	143–398 x 10^3^/µL
D-Dimer	-	>20.00	3.58	1.33	<0.60 µg/mL FEU
Fibrinogen	-	91	132	-	235–490 mg/dL
Heparin Plt Ab	-	Positive	-	-	Negative
Serotonin Release Assay		Positive	-	-	Negative

**Figure 1:**
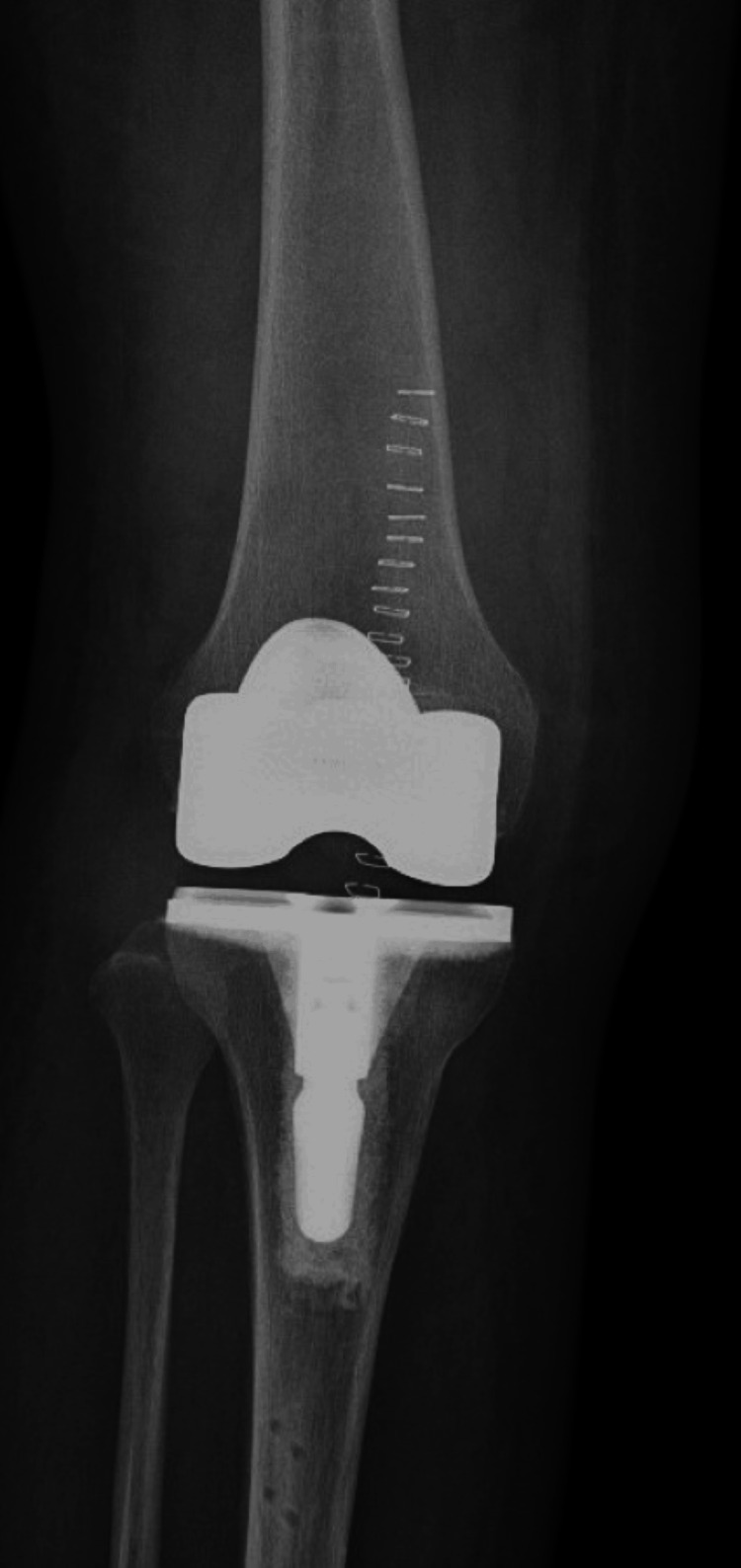
The XR of the right knee showed the presence of a right knee replacement without complication or malalignment, but did show a joint effusion and peritubular soft tissue swelling.

**Figure 2:**
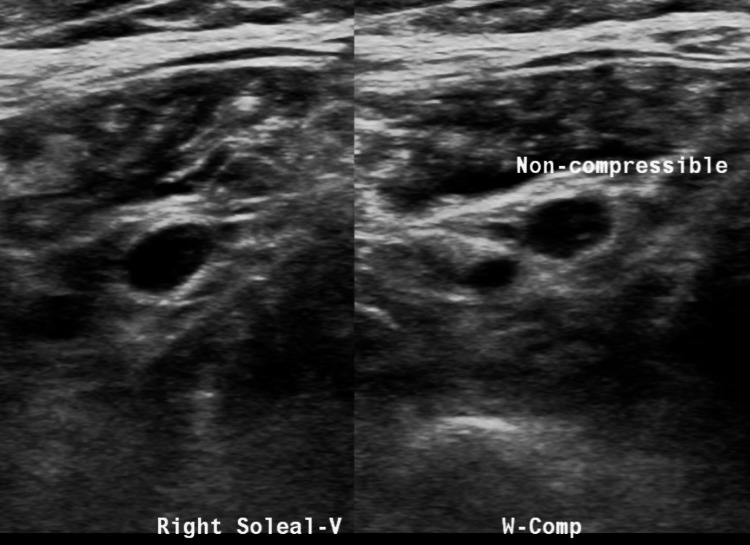
A US duplex lower extremity vein on the right shows evidence of acute/occlusive deep venous thrombosis in the right soleal veins.

**Figure 3:**
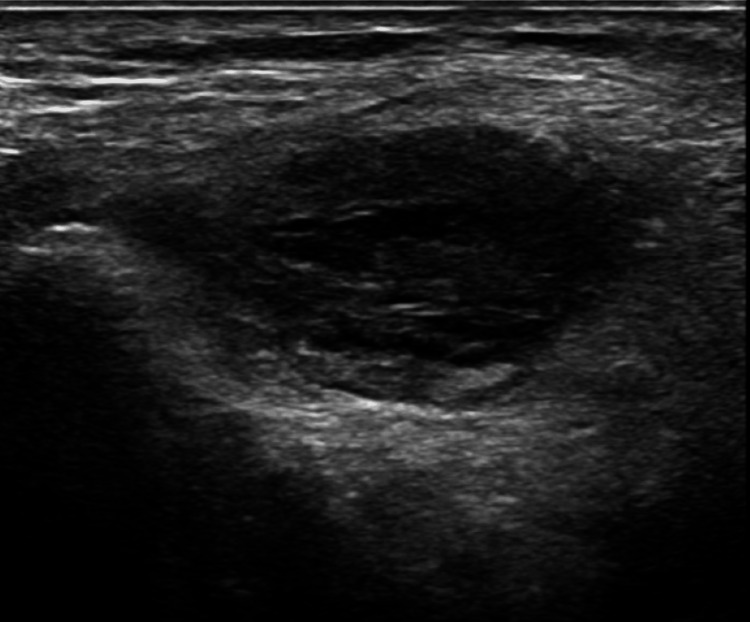
Soft tissue ultrasound of the right lower extremity reveals a posterior knee/superior calf hematoma measuring up to 3.4 cm.

**Figure 4:**
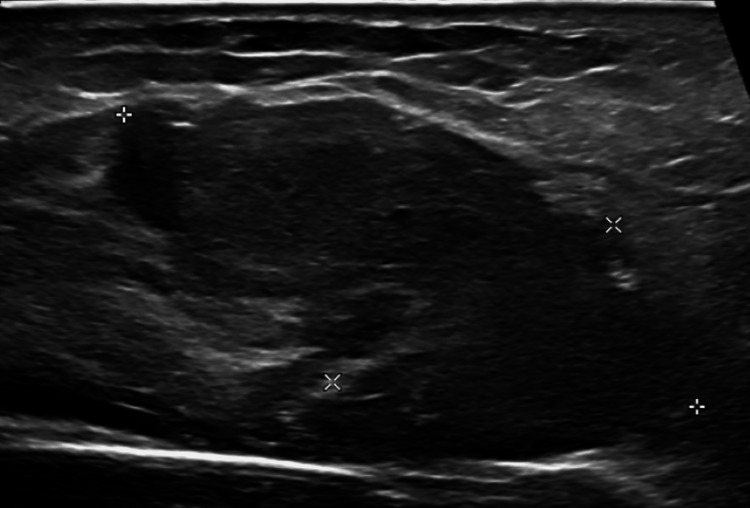
Soft tissue ultrasound of the right lower extremity reveals a lateral knee hematoma measuring up to 4.6 cm.

An X-ray of the right knee showed the presence of a right knee replacement without complications or malalignment, but it did show a joint effusion and peritubular soft tissue swelling (Figure 1). A right lower extremity ultrasound revealed evidence of acute/occlusive deep venous thrombosis in the right soleal veins (Figure 2). Soft tissue ultrasound of the right lower extremity revealed a posterior knee/superior calf hematoma measuring up to 3.4 cm (Figure 3) and a lateral knee hematoma measuring up to 4.6 cm (Figure 4).

Further history revealed that the patient was not exposed to heparin during surgery or in the postoperative course. The hematology-oncology team was consulted. The patient was started on an argatroban drip, which was eventually transitioned to fondaparinux due to the development of a rash on argatroban for the management of autoimmune/spontaneous HIT. The patient was started on dexamethasone 10 mg daily. Her platelet count and D-dimer improved as illustrated in Table 1. She demonstrated no further evidence of bleeding, and the swelling of her right lower extremity improved. Dexamethasone was discontinued, and fondaparinux was transitioned to apixaban. The patient was discharged home.

During follow-up in the hematology-oncology clinic one month later, right leg swelling was noted to have nearly completely resolved. Improvement in hemoglobin and platelet count is noted, as shown in Table 1. She was reported to be tolerating apixaban without evidence of bleeding or additional side effects.

## Discussion

Spontaneous or autoimmune HIT is a rare form of HIT that occurs in the absence of heparin exposure, most commonly in patients with a preceding infection or in those who have undergone a surgical procedure, such as orthopedic surgery. This condition likely results from an autoimmune reaction to endogenous heparins. Diagnostic criteria for this condition include unexplained thrombocytopenia and/or thrombosis without recent heparin exposure and demonstration of anti-PF4 antibodies of the IgG subclass that cause strong in vitro platelet activation in the absence of heparin [5].

A review of medical literature revealed nine previously reported cases of spontaneous HIT in which patients presented with thrombocytopenia and thrombosis in the absence of heparin exposure. Six occurred after orthopedic surgery (knee replacement surgery), two occurred after infection, and one occurred without an identifiable causal event [5-8]. In patients with suspected spontaneous HIT, testing should be performed immediately, and treatment should not be delayed, given the high mortality rate associated with untreated HIT.

## Conclusions

HIT is a life-threatening complication most commonly associated with exposure to heparin. However, in rare cases, HIT has been diagnosed in patients without recent heparin exposure. Most commonly, these patients have had a preceding infection or have undergone a surgical procedure (most commonly orthopedic surgery) before being diagnosed with "spontaneous HIT." Timely recognition of suspected HIT is vital, as early intervention is associated with improved outcomes and a decreased mortality rate.
